# UNMET NEEDS AND PERCEIVED BARRIERS TO ACCESSING HCBS AMONG CAREGIVERS OF VETERANS OF ALL ERAS

**DOI:** 10.1093/geroni/igac059.171

**Published:** 2022-12-20

**Authors:** Ranak Trivedi, Victoria Ngo, Trevor Lee, Marika Humber, Rashmi Risbud, Shreya Desai, Josephine Jacobs, Dolores Gallagher-Thompson

**Affiliations:** Stanford University, Palo Alto, California, United States; VA Palo Alto Health Care System, Menlo Park, California, United States; VA Palo Alto Health Care System, Palo Alto, California, United States; VA Palo Alto Health Care System, Palo Alto, California, United States; University of California, Davis, Davis, California, United States; Veterans Affairs Palo Alto Health Care System, Menlo Park, California, United States; VA Palo Alto Health Care System, Menlo Park, California, United States; Stanford University, Stanford, California, United States

## Abstract

VA has several HCBS to offset caregiver burden, facilitate caregiving, and enhance Veterans’ home-based care, but they remain underutilized. We aimed to describe: the unmet psychosocial and HCBS needs of caregivers, barriers to accessing services, and gaps in available programs. Twenty-three caregivers participated in a 1-hr semi-structured interview (62.9□13.5y; 74.0% women; 47.8% White; 17.4% Hispanic; 65.2% spouses). Caregivers provided 7.3 hrs of daily care (SD=5.5 hrs, Range=1-24); most had provided care for 1+ year. Barriers to accessing HCBS included: a) disagreement with Veterans regarding service preferences and needs; b) lack of awareness of VA and non-VA programs; c) delays in obtaining services; and d) emotional toll of caregiving on personal health and relationship with the Veteran. The VA may need to invest in advertising existing services, develop strategies to match caregivers with available services when needed, and enhancing mental health and relationship quality for Veterans and caregivers.

